# Impact of Diabetes Mellitus on Coronary Artery Disease Severity: A Comparative Analysis of Diabetic and Non-diabetic Patients

**DOI:** 10.7759/cureus.77344

**Published:** 2025-01-12

**Authors:** Muhammad Muneeb Arshad, Muhammad Jalal-ud-Din, Junaid Qayyum, Masooma Zainab, Ibrahim Shah, Saira Fayyaz, Muhammad Sajid

**Affiliations:** 1 Internal Medicine, University Hospitals Birmingham National Health Service (NHS) Foundation Trust, Birmingham, GBR; 2 Internal Medicine, University Hospitals Coventry and Warwickshire, Coventry, GBR; 3 Medicine, District Headquarter (DHQ) Hospital, Rawalpindi, PAK; 4 General Internal Medicine, University Hospitals Coventry and Warwickshire, Coventry, GBR; 5 Cardiology, Gajju Khan Medical College, Swabi, PAK; 6 Cardiology, Sheikh Mohamed Bin Zayed Al Nahyan Institute of Cardiology, Quetta, PAK; 7 Biological Sciences, International Islamic University, Islamabad, PAK

**Keywords:** cad severity, coronary artery disease, diabetes mellitus, fasting blood glucose, hba1c, multi-vessel disease

## Abstract

Background

One of the main risk factors for coronary artery disease (CAD), which is a significant source of morbidity and death, is diabetes mellitus (DM), which also speeds up the disease's course and severity. This study aimed to compare the severity of CAD between diabetic and non-diabetic patients, providing insights to guide clinical decision-making and improve therapeutic outcomes.

Methodology

A prospective, observational study was conducted at the Sheikh Mohamed Bin Zayed Al Nahyan Institute of Cardiology, Quetta, from August 2018 to July 2019, involving 204 adult patients with CAD, consisting of 102 patients with diabetes and 102 without diabetes. Demographic, clinical, and laboratory data were collected, and coronary angiography was performed to assess the severity of CAD, which was defined as a binary outcome variable (1 = severe CAD, 0 = not severe CAD), based on criteria such as multi-vessel involvement and degree of stenosis. Statistical analysis was carried out using IBM SPSS Statistics for Windows, V. 25.0 (IBM Corp., Armonk, NY, USA), with chi-squared tests used for categorical variables (e.g., number of affected vessels, plaque distribution) and independent t-tests for continuous variables (e.g., fasting blood glucose, hemoglobin A1c(HbA1c) levels, lipid profiles). Logistic regression was employed to identify independent predictors of CAD severity, and odds ratios (ORs) with 95% confidence intervals (CI) were calculated, with statistical significance defined as a p-value of less than 0.05.

Results

Diabetic patients exhibited significantly higher rates of multi-vessel disease (70 (68.63%) vs. 52 (50.98%); p = 0.02), three-vessel involvement (37 (36.27%) vs. 25 (24.51%); p = 0.01), and severe stenosis (64 (62.75%) vs. 52 (50.98%); p = 0.019) compared to non-diabetic patients. Fasting blood glucose (158.21 ± 45.63 mg/dL vs. 98.31 ± 12.74 mg/dL; p < 0.001) and HbA1c (8.21 ± 1.34% vs. 5.62 ± 0.41%; p < 0.001) levels were significantly higher in diabetics. Multivariate regression analysis revealed that diabetes (OR = 2.12; 95% CI: 1.35-3.32; p = 0.001), hypertension (OR = 1.45; 95% CI: 1.01-2.08; p = 0.04), elevated glucose levels (fasting blood glucose OR = 1.04; 95% CI: 1.02-1.06; p < 0.001; HbA1c OR = 1.27; 95% CI: 1.15-1.41; p < 0.001), and age (OR = 1.03; 95% CI: 1.01-1.05; p = 0.02) were significant predictors of CAD severity, with each increase in fasting blood glucose, HbA1c, and age associated with higher odds of severe CAD. Other variables, such as gender, hyperlipidemia, smoking history, sedentary lifestyle, and regular exercise, did not show significant associations with CAD severity (p > 0.05). However, a family history of CAD was identified as a significant predictor (OR = 1.56; 95% CI: 1.06-2.31; p = 0.02), suggesting that those with a family history of CAD had 56% higher odds of experiencing severe CAD. Systolic blood pressure (OR = 1.18; 95% CI: 1.07-1.30; p = 0.002) was associated with CAD severity, with each 10 mmHg increase in systolic blood pressure increasing the odds of severe CAD.

Conclusion

DM is linked to more severe CAD, with high blood pressure, HbA1c, and blood glucose levels contributing to increased severity, emphasizing the need for early detection and aggressive treatment to mitigate its impact on heart health, and further research, including long-term follow-up and therapeutic interventions, is needed to enhance management for diabetic CAD patients.

## Introduction

Globally, coronary artery disease (CAD) continues to be a major source of morbidity and death, presenting serious difficulties for healthcare systems [1,2]. Reduced blood flow to the heart muscle is the result of atherosclerotic plaque accumulation, which narrows or blocks coronary arteries in this situation [3]. A sedentary lifestyle, smoking, hypertension, and hyperlipidemia are some of the risk factors that lead to the onset and advancement of CAD [4]. Among them, diabetes mellitus (DM) has become a significant factor in determining the severity of CAD and is often linked to worse clinical results [5].

Diabetes is a metabolic disease characterized by persistently high blood sugar levels brought on by either insulin resistance or insufficiency [6]. It accelerates atherosclerosis and contributes to plaque instability by drastically changing the structure and function of the arteries [7]. Compared to those without diabetes, this mechanism makes diabetics more likely to have serious cardiac events. Additionally, individuals with diabetes often have more diffuse and multi-vessel coronary involvement, which makes treatment plans more difficult and their prognosis poorer [8].

However, although being mediated by a distinct pathophysiological spectrum, non-diabetic patients with CAD still suffer significant risks [9]. Although this group is dominated by classic risk factors like hypertension and dyslipidemia, new research indicates that metabolic and inflammatory pathways may be involved even when diabetes is not present [10,11]. Assessing the degree of CAD in individuals with and without diabetes offers important information on how metabolic diseases worsen coronary pathology and directs specialized treatment approaches [12].

There are still gaps in measuring and comparing the severity of the diseases in these groups, despite significant advancements in our knowledge of the interactions between diabetes and CAD. Enhancing risk segmentation, maximizing management tactics, and lowering unfavorable outcomes all depend on this comparison. By examining these variations, medical professionals and researchers may more effectively handle the particular difficulties that CAD presents to people with and without diabetes.

## Materials and methods

Study design and setting

This prospective, observational study was conducted at the Department of Cardiology, Sheikh Mohamed Bin Zayed Al Nahyan Institute of Cardiology, Quetta, over a duration of one year, from August 2018 to July 2019 after obtaining approval from the institute's Institutional Review Board (approval number: 173/SMBZ-DOC/2018; date: July 2018).

Inclusion and exclusion criteria

Adult patients (18 years of age or older) having a confirmed diagnosis of CAD based on clinical examination and coronary angiography were included in the research. Included were non-diabetic participants without a history of DM and diabetic patients with type 1 or type 2 DM, as defined by conventional criteria (fasting blood glucose ≥126 mg/dL or hemoglobin A1c (HbA1c) ≥6.5%). Only participants who gave their informed permission were taken into account. Patients with a history of percutaneous coronary intervention (PCI) or coronary artery bypass grafting (CABG), acute myocardial infarction or unstable angina at admission, and severe comorbid conditions (e.g., advanced renal failure or terminal cancer), pregnant or lactating women, and patients with incomplete clinical records were excluded.

Sample size

The research had 204 individuals in total, evenly divided into two groups: 102 patients with diabetes (DM group) and 102 patients without diabetes (non-DM group). Participants were chosen from the Department of Cardiology using convenience sampling, taking into account their availability and desire to take part in the research.

Data collection

Clinical exams, diagnostic testing, and patient medical records were used to gather data. Clinical information such as fasting blood glucose, HbA1c levels, lipid profiles, and blood pressure for both diabetic and non-diabetic patients was collected after comprehensive demographic information such as age, gender, medical history, and lifestyle variables. Using coronary angiography, the severity of CAD was evaluated, noting the number of vessels impacted, the degree of stenosis, and other pertinent factors including the kind and location of plaque. After being gathered, all of the data was put into a safe database for further examination.

Statistical analysis

IBM SPSS Statistics for Windows, V. 25.0 (IBM Corp., Armonk, NY, USA), was used for data analysis. The severity of CAD was defined as a binary outcome variable (1 = severe CAD, 0 = not severe CAD), based on criteria such as multi-vessel involvement and the degree of stenosis. To compare the severity of CAD between patients with and without diabetes, chi-squared tests were used for categorical variables (e.g., number of affected vessels, plaque distribution, and CAD severity categories). Independent t-tests were applied for continuous variables (e.g., fasting blood glucose, HbA1c levels, and lipid profiles). Statistical significance was defined as a p-value of less than 0.05. Logistic regression was employed to identify independent predictors of CAD severity, using the binary outcome variable (1 = severe, 0 = not severe), and odds ratios (ORs) with 95% confidence intervals (CI) were calculated.

## Results

The mean age of the 204 patients (102 diabetics and 102 non-diabetics) was 58.43 ± 8.31 years for diabetics and 57.12 ± 7.95 years for non-diabetics. Among diabetics, 60 (58.82%) were male and 42 (41.18%) were female, compared to 55 (53.92%) males and 47 (46.08%) females in the non-diabetic group, showing a comparable gender distribution (Table 1). Diabetics were more likely to have hypertension (80 (78.43%) vs. 68 (66.67%)) and hyperlipidemia (70 (68.63%) vs. 60 (58.82%)), while sedentary lifestyle (60 (58.82%) vs. 58 (56.86%)) and smoking history (45 (44.12%) vs. 50 (49.02%)) were similar between groups. HbA1c levels were markedly higher in diabetics (8.21 ± 1.34% vs. 5.62 ± 0.41%), as was fasting blood glucose (158.21 ± 45.63 mg/dL vs. 98.31 ± 12.74 mg/dL). Diabetics had slightly elevated total cholesterol (213.15 ± 41.25 mg/dL vs. 204.58 ± 39.82 mg/dL), low-density lipoprotein (LDL) cholesterol (130.34 ± 34.74 mg/dL vs. 124.84 ± 31.41 mg/dL), and triglycerides (173.27 ± 45.85 mg/dL vs. 164.51 ± 49.32 mg/dL) compared to non-diabetics, though high-density lipoprotein (HDL) cholesterol was lower (45.79 ± 10.38 mg/dL vs. 48.14 ± 12.43 mg/dL). Blood pressure readings were higher in diabetics, with systolic blood pressure at 141.16 ± 16.23 mmHg compared to 135.47 ± 13.85 mmHg and diastolic blood pressure at 89.23 ± 10.12 mmHg compared to 85.19 ± 9.68 mmHg in non-diabetics.

**Table 1:** Demographic and clinical characteristics of diabetic and non-diabetic patients Statistical significance was defined as a p-value of less than 0.05. CAD: coronary artery disease; HbA1c: hemoglobin A1c; LDL: low-density lipoprotein; HDL: high-density lipoprotein; SD: standard deviation: mg/dL: milligrams per deciliter; mmHg: millimeters of mercury

Characteristic		Diabetic patients (n = 102)	Non-diabetic patients (n = 102)	P-value
Age (years)	Mean ± SD	58.43 ± 8.31	57.12 ± 7.95	0.388
Gender (n; %)	Male	60 (58.82)	55 (53.92)	0.517
	Female	42 (41.18)	47 (46.08)	
Medical history (n; %)	Hypertension	80 (78.43)	68 (66.67)	0.068
	Hyperlipidemia	70 (68.63)	60 (58.82)	0.146
	Smoking history	45 (44.12)	50 (49.02)	0.538
	Family history of CAD	55 (53.92)	52 (50.98)	0.677
Lifestyle factors (n; %)	Sedentary lifestyle	60 (58.82)	58 (56.86)	0.743
	Regular exercise	42 (41.18)	44 (43.14)	0.826
Clinical data (mean ± SD)	Fasting blood glucose (mg/dL)	158.21 ± 45.63	98.31 ± 12.74	<0.001
	HbA1c	8.21 ± 1.34	5.62 ± 0.41	<0.001
	Total cholesterol (mg/dL)	213.15 ± 41.25	204.58 ± 39.82	0.193
	LDL cholesterol (mg/dL)	130.34 ± 34.74	124.84 ± 31.41	0.268
	HDL cholesterol (mg/dL)	45.79 ± 10.38	48.14 ± 12.43	0.235
	Triglycerides (mg/dL)	173.27 ± 45.85	164.51 ± 49.32	0.196
	Systolic blood pressure (mmHg)	141.16 ± 16.23	135.47 ± 13.85	0.014
	Diastolic blood pressure (mmHg)	89.23 ± 10.12	85.19 ± 9.68	0.029

Patients with diabetes exhibited significantly different levels of CAD severity compared to non-diabetic patients (Table 2). Diabetic patients were more likely to have multi-vessel involvement, with 70 (68.63%) compared to 52 (50.98%) non-diabetic patients (p = 0.02; χ² = 5.31). Single-vessel involvement was less common in diabetic patients (32 (31.37%) vs. 50 (49.02%); p = 0.322; χ² = 4.68). Regarding the number of affected vessels, 25 (24.51%) diabetic patients had one vessel affected, compared to 42 (41.18%) non-diabetic patients (p = 0.07; χ² = 3.344), while 40 (39.22%) diabetic patients and 35 (34.31%) non-diabetic patients had two vessels affected (p = 0.63; χ² = 0.51). Three-vessel disease was more prevalent among diabetics, with 37 (36.27%) compared to 25 (24.51%) non-diabetic patients (p = 0.01; χ² = 6.62).

**Table 2:** Comparison of CAD severity between diabetic and non-diabetic patients Statistical significance was defined as a p-value of less than 0.05. OR: odds ratio; CI: confidence interval; DM: diabetes mellitus; CAD: coronary artery disease; HbA1c: hemoglobin A1c; mg/dL: milligrams per deciliter; mmHg: millimeters of mercury; χ²:chi-squared value;t: t-test statistic;df: degrees of freedom;€: independent t-test;*: chi-squared test

CAD severity parameter		Diabetic patients (n = 102)	Non-diabetic patients (n = 102)	P-value	Statistic (χ² or t)	df
Number of affected vessels	1 vessel affected	25 (24.51%)	42 (41.18%)	0.07*	5.42	1
	2 vessels affected	40 (39.22%)	35 (34.31%)	0.63*	0.51	1
	3 vessels affected	37 (36.27%)	25 (24.51%)	0.01*	6.62	1
Degree of stenosis	Mild (1-49%)	10 (9.80%)	20 (19.61%)	0.014*	3.81	1
	Moderate (50-69%)	28 (27.45%)	30 (29.41%)	0.775*	0.05	1
	Severe (≥70%)	64 (62.75%)	52 (50.98%)	0.019*	2.57	1
Plaque distribution	Single-vessel involvement	32 (31.37%)	50 (49.02%)	0.322*	4.68	1
	Multi-vessel involvement	70 (68.63%)	52 (50.98%)	0.02*	5.31	1
Plaque type	Stable plaque	30 (29.41%)	40 (39.22%)	0.076*	3.239	1
	Unstable plaque	72 (70.59%)	62 (60.78%)			
Continuous variables	Fasting blood glucose (mg/dL)	158.21 ± 45.63	98.31 ± 12.74	<0.001€	10.43	202
	HbA1c	8.21 ± 1.34	5.62 ± 0.41	<0.001€	18.45	202
	Total cholesterol (mg/dL)	213.15 ± 41.25	204.58 ± 39.82	0.29€	1.06	202
	LDL cholesterol (mg/dL)	130.34 ± 34.74	124.84 ± 31.41	0.32€	0.99	202
	HDL cholesterol (mg/dL)	45.79 ± 10.38	48.14 ± 12.43	0.34€	0.96	202
	Triglycerides (mg/dL)	173.27 ± 45.85	164.51 ± 49.32	0.24€	1.16	202
	Systolic blood pressure (mmHg)	141.16 ± 16.23	135.47 ± 13.85	0.02€	2.39	202
	Diastolic blood pressure (mmHg)	89.23 ± 10.12	85.19 ± 9.68	0.04€	2.08	202

The degree of stenosis also varied significantly. Severe stenosis (≥70%) was more common in diabetics (64 (62.75%) vs. 52 (50.98%); p = 0.019; χ² = 5.439). Mild stenosis (1-49%) was less frequent in diabetic patients (10 (9.80%) vs. 20 (19.61%); p = 0.014; χ² = 3.81), while moderate stenosis (50-69%) was comparable between both groups (28 (27.45%) vs. 30 (29.41%); p = 0.775; χ² = 0.05). In terms of plaque type, unstable plaques were more common in diabetic patients (72 (70.59%) vs. 62 (60.78%); p = 0.076; χ² = 3.239), while stable plaques were more common in non-diabetic patients (40 (39.22%) vs. 30 (29.41%); p = 0.076; χ² = 3.239).

Regarding continuous variables, diabetic patients had significantly higher fasting blood glucose levels (158.21 ± 45.63 mg/dL vs. 98.31 ± 12.74 mg/dL; p < 0.001; t = 10.43) and higher HbA1c values (8.21 ± 1.34% vs. 5.62 ± 0.41%; p < 0.001; t = 18.45). Systolic blood pressure was significantly higher in diabetic patients (141.16 ± 16.23 mmHg vs. 135.47 ± 13.85 mmHg; p = 0.02; t = 2.39), as was diastolic blood pressure (89.23 ± 10.12 mmHg vs. 85.19 ± 9.68 mmHg; p = 0.04; t = 2.08). Total cholesterol, LDL cholesterol, HDL cholesterol, and triglycerides did not show significant differences between the two groups (all p > 0.05).

A number of significant predictors of the severity of CAD were found by the multivariate regression analysis (Table 3). Significant correlations were found between increased CAD severity and DM (OR = 2.12; p = 0.001), age (OR = 1.03 per year increase; p = 0.02), hypertension (OR = 1.45; p = 0.04), family history of CAD (OR = 1.56; p = 0.02), fasting blood glucose (OR = 1.04 per 10 mg/dL increase; p < 0.001), HbA1c (OR = 1.27 per 1% increase; p < 0.001), and systolic blood pressure (OR = 1.18 per 10 mmHg increase; p = 0.002).

**Table 3:** Multivariate regression analysis of predictors of CAD severity DM: diabetes mellitus; CAD: coronary artery disease; HbA1c: hemoglobin A1c; OR: odds ratio; CI: confidence interval; mg/dL: milligrams per deciliter; mmHg: millimeters of mercury

Variable	OR	95% CI	P-value
Diabetes (DM vs. non-DM)	2.12	1.35-3.32	0.001
Age (per year increase)	1.03	1.01-1.05	0.02
Gender	1.25	0.83-1.87	0.27
Hypertension	1.45	1.01-2.08	0.04
Hyperlipidemia	1.32	0.91-1.91	0.14
Smoking history	1.17	0.79-1.73	0.44
Family history of CAD	1.56	1.06-2.31	0.02
Sedentary lifestyle	1.15	0.76-1.74	0.52
Regular exercise	0.86	0.57-1.29	0.46
Fasting blood glucose (per 10 mg/dL increase)	1.04	1.02-1.06	<0.001
HbA1c (per 1% increase)	1.27	1.15-1.41	<0.001
Systolic blood pressure (per 10 mmHg increase)	1.18	1.07-1.30	0.002

## Discussion

DM is acknowledged as a significant risk factor that contributes to the severity of CAD, which is still a major global health problem. Significant variations in the degree of coronary involvement were found between the two groups in this investigation, which sought to assess the severity of CAD in individuals with and without diabetes. With a greater percentage of multi-vessel disease (68.63% vs. 50.98%; p = 0.02), three-vessel involvement (36.27% vs. 24.51%; p = 0.01), and severe stenosis (62.75% vs. 50.98%; p = 0.019), the results demonstrated that diabetic individuals had more severe CAD than non-diabetics. These findings are in line with earlier research showing a link between diabetes and greater involvement of the coronary arteries [13]. For example, our results of increased multi-vessel disease in diabetics are consistent with research by Jung et al. that found diabetic patients are more likely to develop multi-vessel CAD [14].

In this study, diabetic patients exhibited significantly higher rates of multi-vessel disease (68.63% vs. 50.98%; p = 0.02), three-vessel involvement (36.27% vs. 24.51%; p = 0.01), and severe stenosis (62.75% vs. 50.98%; p = 0.019) compared to non-diabetic patients. Diabetics also had higher levels of fasting blood glucose (158.21 ± 45.63 mg/dL vs. 98.31 ± 12.74 mg/dL; p < 0.001) and HbA1c (8.21 ± 1.34% vs. 5.62 ± 0.41%; p < 0.001). Moreover, lipid abnormalities were observed, but they were not statistically significant. Diabetic patients had slightly higher total cholesterol (213.15 ± 41.25 mg/dL vs. 204.58 ± 39.82 mg/dL; p = 0.29), higher LDL cholesterol (130.34 ± 34.74 mg/dL vs. 124.84 ± 31.41 mg/dL; p = 0.32), lower HDL cholesterol (45.79 ± 10.38 mg/dL vs. 48.14 ± 12.43 mg/dL; p = 0.34), and slightly higher triglycerides (173.27 ± 45.85 mg/dL vs. 164.51 ± 49.32 mg/dL; p = 0.24). Additionally, diabetics were more likely to have elevated systolic blood pressure (141.16 ± 16.23 mmHg vs. 135.47 ± 13.85 mmHg; p = 0.02) and diastolic blood pressure (89.23 ± 10.12 mmHg vs. 85.19 ± 9.68 mmHg; p = 0.04), contributing to the increased severity of CAD. Another important finding was the higher prevalence of unstable plaques in diabetics (70.59% vs. 60.78%), indicating that diabetes increases the risk of severe cardiovascular events by accelerating plaque instability. These results are consistent with other studies that suggest diabetes increases the risk of plaque rupture due to increased inflammation and altered vascular function [15]. Together, these factors, including lipid abnormalities, high blood pressure, and unstable plaques, contribute to more severe CAD in diabetic patients.

Patients with diabetes had substantially higher fasting blood glucose (158.21 ± 45.63 mg/dL vs. 98.31 ± 12.74 mg/dL; p < 0.001) and HbA1c (8.21 ± 1.34% vs. 5.62 ± 0.41%; p < 0.001) levels, which supports earlier research that linked hyperglycemia to more severe CAD. Elevated glucose levels were linked to increased plaque load and coronary artery stenosis, according to a research by Suzuki et al. [16]. Our findings support previous findings, suggesting that elevated glucose levels play a role in the accelerated atherosclerosis process seen in diabetics.

The established idea that diabetes and hypertension are independent risk factors for severe CAD is further supported by the multivariate regression analysis, which found several significant predictors of CAD severity, including DM (OR = 2.12; p = 0.001), hypertension (OR = 1.45; p = 0.04), and elevated fasting blood glucose (OR = 1.04 per 10 mg/dL increase; p < 0.001). These results are in line with earlier research that showed the substantial influence of hypertension and hyperglycemia on the severity of CAD [14,17].

The primary strength of this study lies in its prospective design and the comprehensive evaluation of CAD severity using both clinical and angiographic parameters, enabling a robust comparison between diabetic and non-diabetic patients. The use of multivariate regression analysis to account for confounding variables adds further rigor to the findings. However, the study has several limitations. Convenience sampling may introduce selection bias, and the single-center design limits the generalizability of the results to broader populations. Excluding patients with prior revascularization, severe comorbidities, and incomplete clinical records might have excluded more complex cases of CAD, potentially underestimating disease severity in real-world settings. The observational nature of the study prevents causal inferences, and the reliance on historical and self-reported data for lifestyle factors could introduce recall bias.

## Conclusions

This study demonstrates that DM significantly exacerbates the severity of CAD, with diabetic patients exhibiting more advanced coronary involvement, higher rates of multi-vessel disease, and greater plaque instability compared to non-diabetic patients. High blood pressure, HbA1c, and blood glucose levels were shown to be important factors in the increased severity of CAD in diabetics. These results highlight how crucial it is to detect diabetes early and treat it aggressively in order to lessen its negative effects on heart health. To further understand the processes behind these disparities and to improve treatment approaches for diabetic individuals with CAD, more study is required, including long-term follow-up and examination of therapeutic treatments.
